# A systematic review of the clinical presentation, treatment and relapse characteristics of human *Plasmodium ovale* malaria

**DOI:** 10.1186/s12936-017-1759-2

**Published:** 2017-03-11

**Authors:** Mirjam Groger, Hannah S. Fischer, Luzia Veletzky, Albert Lalremruata, Michael Ramharter

**Affiliations:** 1 Division of Infectious Diseases and Tropical Medicine, Department of Medicine I, Medical University of Vienna, Vienna, Austria; 2 Centre de Recherches Médicales de Lambaréné, Lambaréné, Gabon; 3 Institut für Tropenmedizin, Universität Tübingen, Tübingen, Germany

**Keywords:** *Plasmodium ovale*, Treatment evaluation, Relapse characteristics, Severe *Plasmodium ovale* malaria, Congenital *Plasmodium ovale* malaria

## Abstract

**Background:**

Despite increased efforts to control and ultimately eradicate human malaria, *Plasmodium ovale* malaria is for the most part outside the focus of research or public health programmes. Importantly, the understanding of *P. ovale*—nowadays regarded as the two distinct species *P. ovale wallikeri* and *P. ovale curtisi*—largely stems from case reports and case series lacking study designs providing high quality evidence. Consecutively, there is a lack of systematic evaluation of the clinical presentation, appropriate treatment and relapse characteristics of *P. ovale* malaria. The aim of this systematic review is to provide a systematic appraisal of the current evidence for severe manifestations, relapse characteristics and treatment options for human *P. ovale* malaria.

**Methods and results:**

This systematic review was performed according to the PRISMA guidelines and registered in the international prospective register for systematic reviews (PROSPERO 2016:CRD42016039214). *P. ovale* mono-infection was a strict inclusion criterion. Of 3454 articles identified by the literature search, 33 articles published between 1922 and 2015 met the inclusion criteria. These articles did not include randomized controlled trials. Five prospective uncontrolled clinical trials were performed on a total of 58 participants. *P. ovale* was sensitive to all tested drugs within the follow-up periods and on interpretable in vitro assays. Since its first description in 1922, only 18 relapsing cases of *P. ovale* with a total of 28 relapse events were identified in the scientific literature. There was however no molecular evidence for a causal relationship between dormant liver stages and subsequent relapses. A total of 22 severe cases of *P. ovale* malaria were published out of which five were fatal. Additionally, two cases of congenital *P. ovale* malaria were reported.

**Conclusions:**

Current knowledge of *P. ovale* malaria is based on small trials with minor impact, case reports and clinical observations. This systematic review highlights that *P. ovale* is capable of causing severe disease, severe congenital malaria and may even lead to death. Evidence for relapses in patients with *P. ovale* malaria adds up to only a handful of cases. Nearly 100 years after *P. ovale’*s first description by Stephens the evidence for the clinical characteristics, relapse potential and optimal treatments for *P. ovale* malaria is still scarce.

**Electronic supplementary material:**

The online version of this article (doi:10.1186/s12936-017-1759-2) contains supplementary material, which is available to authorized users.

## Background

In 2015, 214 million new cases of clinical malaria accounting for around 438,000 deaths were identified worldwide [1]. Although these numbers are decreasing, they remain striking, as most were preventable. Malaria is among the “big three” infectious diseases and receives relatively much attention and funding. However, research focuses almost entirely on the most prevalent malaria parasite *Plasmodium falciparum*, whereas the other Plasmodium species are widely neglected.

*Plasmodium ovale* has so far received comparatively little attention in medical research. The primary focus after its first description by Stephens in 1922 was the characterization of its microscopic morphology [2–4]. Interestingly, it has been demonstrated recently by molecular methods that *P. ovale* essentially consists of two distinct sympatric species termed *P. ovale curtisi* and *P. ovale wallikeri* [5]. So far, only few clinical, epidemiological and therapeutic studies report specific data for *P. ovale* subspecies. However, based on molecular analysis the geographic distribution of *P. ovale* seems larger than previously thought [6, 7].

Although considered to induce only mild disease of minor importance, case reports indicate its potential of evoking severe disease and even death [8, 9]. A systematic evaluation of potential complications of *P. ovale* malaria is currently missing. Treatment of *P. ovale* was historically developed based on the empiric use of anti-malarial drugs administered for *P. falciparum* and *Plasmodium vivax* malaria. Since then, no systematic drug evaluation or development programme has been undertaken for *P. ovale* malaria.

One of the cornerstones of today’s understanding of ovale malaria is its potential to lead to hypnozoite induced relapse. This feature of tertian malaria is the reason for recommending the use of the antihypnozoite drug primaquine in *P. ovale* infections. Interestingly, this concept has been challenged recently based on a lack of experimental and clinical data supporting the hypnozoite model in ovale malaria [10–12].

These important gaps in the perception of the basic biology of *P. ovale*, of the potential to cause severe disease, of the evidence behind current treatment recommendations and of its potential to cause relapse were the principal reasons to endeavor for a systematic evaluation of all available evidence of *P. ovale* research since its description in 1922.

## Methods

This systematic review was conducted following the PRISMA guidelines [13]. The protocol was registered at the international prospective register of systematic reviews (PROSPERO 2016:CRD42016039214). The scientific databases MEDLINE, EMBASE, Cochrane Library, Scopus, CINAHL, Conference Proceedings Citation Index, Web of Science/Science Citation Index Expanded and DARE were searched for publications between 1922 and 2015 using “*P. ovale*” as search term. Additionally, Google Scholar was searched for publications between 1922 and 1971 to increase the coverage for the pre-internet era. Furthermore, ClinicalTrials.gov and the EU Clinical Trials Register were checked for unpublished studies on *P. ovale*.

### Data extraction

Screening, selection and data extraction were performed independently by the first and the second author. Disagreements and uncertainties at any stage of the process were discussed and resolved by consensus. If needed, the last author was consulted for a final decision. Only English, German and French articles were included in this analysis unless there was clear indication for relevant information of publications in other languages. Full texts of potentially relevant articles were obtained and articles from other sources were included in the pool of articles. Articles were matched to three different categories: treatment, severity and relapse.

*Plasmodium ovale* mono-infection of a human subject, defined by diagnosis based on microscopy and/or polymerase chain reaction (PCR) was a strict eligibility criterion for all categories. Additionally, separate definitions as follows were applicable for each category. Case reports were not used for treatment evaluation, otherwise there were no inclusion restrictions regarding types of studies. For in vitro studies, only assays with interpretable results were considered. Severe *P. ovale* malaria was determined on the basis of the 2014 WHO criteria for severe falciparum malaria [14] and other serious or life threatening clinical conditions as defined by the authors. As parasite counts are generally lower in severe *P. ovale* than in severe *P. falciparum* malaria [7] no threshold was determined for parasitaemia. A relapse was defined as a reappearing *P. ovale* parasitaemia following an initially diagnosed and adequately treated *P. ovale* “primary infection” (regardless of 8-aminoquinoline application) and subsequent permanent residence in a non-endemic country. The term “primary infection” was used to describe the first reported *P. ovale* infection in the article, which was adequately treated (important for malaria infection studies, where patients were mostly not treated in case of self-limiting infection). The period between primary infection and relapse and two relapse events, respectively, was counted as time between the date of treatment and the first mentioned date of reappearance. In order not to confuse delayed primary attacks with relapses, articles where the primary infection was not explicitly stated to have been a *P. ovale* infection were excluded.

### Outcomes

Primary outcomes were adequate clinical and parasitological response on day 28, frequency of severe complications and the number of reported true relapses. Secondary outcomes were to obtain relapse characteristics, treatment regimen used, parasite clearance time (PCT), fever clearance time (FCT) and treatment outcome.

### Data synthesis and risk of bias assessment

References were compiled in EndNote X6 (Thomson Reuters) and extracted data was collected in a standardized Microsoft^®^ Excel^®^ 2013 datasheet. Descriptive statistics were performed using IBM^®^ SPSS^®^ Statistics 20. Applicable risk of bias was assessed in applicable studies using the Cochrane collaboration’s tool for assessing risk of bias in combination with the methods guide for comparative effectiveness reviews [15, 16]. To assess overall quality of reporting an evaluation tool was created uniformly for all included study designs following the study quality assessment of the case series studies tool of the National Institute of Health [17].

## Results

### Study selection

**Fig. 1:**
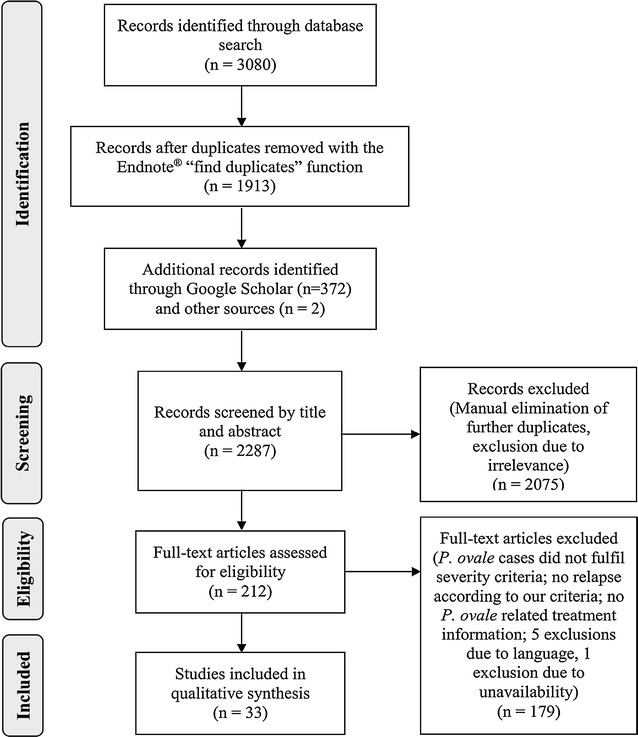
PRISMA [13] adapted flow diagram

The search yielded 3454 publications. After elimination of duplicates and screening of available titles and abstracts for relevance, 212 articles were selected for full review (Fig. 1). Two articles were added from other sources and a total of 36 articles met the required criteria. Of this pool, two articles reporting severe cases were excluded due to incomplete data and one because of double reporting, leaving 33 articles for data extraction.

No report was rated as having a low risk of bias due to the underlying study design. There were many case reports which made the systematic review especially vulnerable to selection bias and publication bias. To deal with publication bias, results from Clinical Trial Registers were included. As data were not used for a meta-analysis, missing data items did not influence individual risk of bias assessment. Individual risk of bias within studies as well as completeness of reporting are given in the Additional files 1, 2 and 3.

### Study population

The study population of the included articles was heterogeneous. It consisted of residents in malaria-endemic areas, individuals visiting friends or relatives (VFRs), tourists, professionals temporarily residing in endemic countries, neurosyphilis patients treated with iatrogenic malaria infection, experimental malaria infections and one case of malaria transmission by blood transfusion. Study designs were diverse, but lacked designs judged to provide high quality evidence. There were no randomized controlled clinical trials (RCTs) and small sample size case series and case reports dominated the findings. The majority of reports did not distinguish between the two sympatric *P. ovale* species.

Not all endemic areas of the world were represented by the data that was found. The majority of cases was from sub-Saharan Africa. Asia was represented by Indonesia, Papua New Guinea and India. There were no eligible *P. ovale* reports from South America. Detailed information about treatment, severe disease and relapse is subsequently described.

### Treatment

The literature search yielded five prospective studies evaluating treatment for *P. ovale* in a total of 58 participants. Baseline characteristics are outlined in Table 1. One study was conducted in Indonesia [18], two in Cameroon [19, 20], one in Gabon [21] and one in France on returnees from sub-Saharan Africa [22]. One trial was exclusively designed for *P. ovale* infected individuals [19]. Artesunate, atovaquone, chloroquine, mefloquine and pyronaridine were used as study drugs. Two prospective clinical trials with 13 participants in total chose chloroquine as study drugs [18, 21]. In general, sample sizes were small and control groups were missing in all 5 prospective studies. In fact, the largest study recruiting 30 patients evaluated artesunate therapy. Although the authors classified it as randomized trial, neither a placebo group nor a second treatment arm were described [19].

**Table 1:** Baseline characteristics in prospective uncontrolled clinical trials

Authors	No of *P. ovale* cases	Age (years)	Sex	Patients’ status	Origin of infection
Siswantoro et al. [18]	11	28 (median)	8 M, 3 F	R	Indonesia
Same-Ekobo et al. [19]	30	–	–	R	Cameroon
Ringwald et al. [20]	8	17 (median)	–	R	Cameroon
	2	8 (median)	–	R	Cameroon
Radloff et al. [21]	3	>10 years	–	R	Gabon
Danis et al. [23]	4	19–32	–	T	Sub-Saharan Africa

*No* number, *M* male, *F* female, *R* resident, *T* tourist, – not mentioned in the original publication

The longest follow-up period was 28 days, therefore, treatment success could not be obtained for days 42 and 63. Besides Siswantoro et al. (eight male, three female) [18], no publication reported the participants’ sex. For further details see Table 2. Two clinical trials additionally performed in vitro drug sensitivity testing. Interpretable assays showed no resistances of *P. ovale* against amodiaquine, artesunate, chloroquine, mefloquine, piperaquine or pyronaridine [18, 20].

**Table 2:** Treatment characteristics

Authors	Diagnostics	Parasitaemia	Drug	Dosing (total)	Period	Adverse events	Mean FCT (h)	Mean PCT (h)	Cure	Last day of observation
Siswantoro et al.	MIC + PCR	645 p/µl	Chloroquine	25 mg/kg (+150 mg base)	Over 3 days	–	24	48	Y~	28
Same-Ekobo et al.	MIC	534,642 p/µl (total)	Artesunate	600 mg	Over 5 days	Vertigo, non-severe transient decrease of reticulocytes in 1 participant	36.6	38.8	Y	14
Ringwald et al.	MIC	2250–40,680 p/µl	Pyronaridine	32 mg/kg	Over 3 days	Mild gastrointestinal disturbances, pruritus	45	57	Y	14
	MIC	6656–13,680 p/µl	Chloroquine	25 mg/kg	Over 3 days	–	24	60	Y	14
Radloff et al.	MIC	700–5000 p/µl	Atovaquone/proguanil	3000 mg/1200 mg	Over 3 days	–	–	72–168	Y	28
Danis et al.	–	–	Mefloquine	0.5–1.25 mg	Once and twice	–	–	72–120	Y	–

*MIC* microscopy, *PCR* polymerase chain reaction, *Y* yes, –not mentioned in the original publication, *~* adequate clinical and parasitological response of *P. ovale* at day 28, however, reappearing *P. vivax* in follow up period at days 14 and 23

### Description of complicated and severe *P. ovale* malaria

Twenty two cases of severe *P. ovale* malaria were identified in scientific literature. Nigeria was the most commonly reported place of potential infection in travel histories (4 times) followed by Ghana, Cameroon and the Democratic Republic of Congo (3 times), and Ivory Coast and Niger (twice). The only non-African country reporting a complicated disease course was India (once). Mean age was 35.8 ± 13.6 years standard deviation (SD), with a range from 17 to 75 years. Fourteen cases were male, six female, for two sex was unknown. In Table 3, baseline characteristics are displayed in more detail. In 15 cases, *P. ovale* was diagnosed by microscopy. Seven patients were diagnosed by microscopy and PCR, out of which 2 cases were microscopically negative with a positive PCR result [24]. Species specific PCR was performed for four cases. Two were positive for *P. ovale curtisi* [8, 24], 1 for *P. ovale wallikeri* and for 1 species differentiation could not be deducted from the article [24, 25].

**Table 3:** Baseline characteristics of severely diseased *P. ovale* cases

Authors, year of publication	Age	Sex	Patient status	Travel history	Chemoprophylaxis
Tomar et al. [61], 2015	75	M	R	None, resident of India	NA
Lemmerer et al. [62], 2015	29	M	W	Democratic Republic of Congo	–
Strydom et al. [36], 2014	42	M	W	Guinea, Mozambique	None
Rojo-Marcos et al. [24], 2014	17	F	–	–	–
	31	M	–	–	–
Lau et al. [8], 2013	59	M	T	Nigeria	Mefloquine
Hachimi et al. [42], 2013	31	M	–	Democratic Republic of Congo	–
Lahlou et al. [41], 2012	28	M	W	Democratic Republic of Congo	–
Roze et al. [63], 2011	24	M	W	Chad, Ivory Coast	Doxycycline
Coton et al. [64], 2011	33	M	W	Djibouti	–
Haydoura et al. [65], 2010	46	F	B	NA	NA
Cinquetti [66], 2010	34	M	W	Ivory Coast, Senegal	Doxycycline
Rojo-Marcos et al. [25], 2008	43	M	V	Nigeria	None
Rubinstein et al. [67], 2005	23	M	–	Nigeria	–
Filler et al. [68], 2003	39	F	T	Cameroon, Botswana, Zimbabwe, South Africa	Yes, drug unknown
Lee et al. [69], 1999	31	F	T	Ghana	Mefloquine
Patel [70], 1993	42	M	T	Central and southern Africa	NA
Facer et al. [9], 1991	51	F	T	Ghana	None
Monlun, et al. [71], 1989	38	M	T	Niger	Chloroquine
Bock [72], 1939	23	–	W	Western Africa, Cameroon	Chinoplasmine
	20	–	W	Western Africa, Cameroon	Quinine (irregular)
Fairley [73], 1933	28	M	T	Nigeria, Ghana, Gambia, Sierra Leone	Quinine

*M* male, *F*, female, *T* tourist, *R* resident, *W* work, *B* blood transfusion, *V* visiting friends or relatives, *NA* not applicable, – not mentioned in the original publication

For the 22 patients with severe clinical conditions, 15 different features of severity could be identified. Taking the patients together, 35 severe conditions were reported. Acute respiratory distress syndrome (ARDS) was reported in five patients and therefore was the most prevalent severe condition. It was followed by anaemia with a hemoglobin level <7 g/dl, and pulmonary edema which occurred in 4 patients. 5 of the reported cases died and 3 patients had organic sequelae, however, 64% of the reported cases (n = 14) survived without sequelae. The majority of deaths occurred following onset of ARDS. Further details are displayed in Table 4.

**Table 4:** Characteristics of severe *P. ovale* disease

Authors	Diagnostics	Parasitaemia	Body temperature (°C)	Features of severity	Treatment	Concomitant medication	Outcome	Comment
Tomar et al. [61]	MIC + PCR	–	39	Bilirubin >50 µm/l, creatinine >265 µm/l, systolic blood pressure <80 mmHg, hemoglobinuria	Artesunate iv	Ceftriaxone iv, antipyretics	Survival without sequelae	
Lemmerer et al. [62]	MIC	–	40.5	Splenic rupture	Chloroquine, 2325 mg po over 2 days	–	Survival with sequelae	
Strydom et al. [36]	MIC + PCR	1.4%	39.5	Bilirubin >50 µm/l, systolic blood pressure <80 mmHg	Quinine, 600 mg iv eight hourly and doxycycline 100 mg twelve hourly	Ceftriaxone	Survival without sequelae	
Rojo-Marcos et al. [24]	MIC + PCR (microscopy neg, PCR positive)	Neg^a^	–	Hemoglobin <7 g/dl^a^	–	–	Survival without sequelae^a^	
	MIC + PCR (microscopy neg, PCR positive)	Neg^a^	–	Hemoglobin <7 g/dl^a^	–	–	Survival without sequelae^a^	
Lau et al. [8]	MIC + PCR	0.18%	40.8	Creatinine >265 µm/l, acidosis, ARDS	Chloroquine 150 mg base for 2 days, quinine for 1 day and artesunate for 7 days	Ceftriaxone, piperacillin/tazobactam, vancomycin, imipenem, meropenem	Death	The first antibiotic was started on day 4 despite negative blood cultures; *Enterobacter cloacae* was found on day 15 (nosocomial sepsis); seizures were described on day 17; atrial fibrillation on day 22; asystole on day 32
Hachimi et al. [42]	MIC	<0.2%	39	ARDS	Quinine	–	Death	History of tuberculosis 11 years ago
Lahlou et al. [41]	MIC	0.2%	–	ARDS	Quinine	Ciprofloxacin	Death	History of treated pulmonary tuberculosis 10 years ago
Roze et al. [63]	MIC	0.2%	–	ARDS	Chloroquine and quinine	–	Survival without sequelae	History of tuberous sclerosis and spontaneous pneumothorax
Coton et al. [64]	MIC	–	40	Acute pericarditis	Chloroquine 30 mg/kg over 6 days	Ketoprophen, omeprazol, aspirin	Survival without sequelae	
Haydoura et al. [65]	MIC	1.1%	40	Oxygen saturation <92%, ARDS	Quinine iv for 7 days and doxycycline po for 7 days	Warfarin	Survival without sequelae	History of MTHFR, secondary portal vein thrombosis and severe lower gastrointestinal bleeding from hemorrhoids requiring 7 units of red blood cells
Cinquetti [66]	MIC + PCR	0.001%	39.5	Splenic infarction	Quinine 8 mg/kg iv three times daily	–	Survival with sequelae	
Rojo-Marcos et al. [25]	MIC + PCR	6000 trophozoites + gametocytes/µl	40.5	ARDS	Chloroquine for 3 days	–	Survival without sequelae	History of diabetes mellitus and hypertension; incomplete left bundle block in the predose ECG followed by left ventricular hypertrophy in the day 30 ECG; presence of Mansonella perstans; nosocomial Acinetobacter baumanii in broncho-alveolar aspirate
Rubinstein et al. [67]	MIC	0.2%	–	Bilirubin >50 µm/l	Quinine for 7 days and doxycycline for 7 days	–	Survival without sequelae	
Filler et al. [68]	MIC	–	–	Hemoglobin <7 g/dl, splenic rupture, cardiac arrest	Quinine sulfate and doxycycline, followed by quinidine iv	–	Death	History notably of MS
Lee et al. [69]	MIC	0.1%	39	Oxygen saturation <92%, pulmonary edema	Chloroquine	–	Survival without sequelae	Negative blood cultures
Patel [70]	MIC	–	–	Splenic rupture	Chloroquine	–	Survival with sequelae	
Facer et al. [9]	MIC	1.8%	NA	Splenic rupture	NA	NA	Death	Diagnosis post mortem; absence of *P. falciparum* confirmed with “DNA analysis”
Monlun et al. [71]	MIC	–	41	Cardiomyopathy	Chloroquine 500 mg/day for 5 days	–	Survival without sequelae	Cardiomyopathy resolved without additional specific treatment
Bock [72]	MIC	–	–	Hemoglobin <7 g/dl	Mepacrine	–	Survival without sequelae	Case 2
	MIC	–	40	Cardiac arrhythmia	Mepacrine	–	Survival without sequelae	Case 15
Fairley [73]	MIC	–	38.3	Hemoglobinuria	Mepacrine 0.1 g three times daily for 6 days and quinine bihydrochloride 7.5 g/day iv for 5 days	Saline	Survival without sequelae	

*MIC* microscopy, *PCR* polymerase chain reaction, *po* per os, *iv* intravenous, *NA* not applicable, *ARDS* acute respiratory distress syndrome, *MTHFR* methylentetrahydrofolate reductase defect, *MS* multiple sclerosis, *–* not mentioned in the original publication

^a^Information provided by the author

### Congenital malaria

Besides the clinically severe cases described above, two independent cases of congenital *P. ovale* malaria were identified presenting with severe anaemia [26, 27]. The two mothers (both secundigravidae) had resided in an African country prior to birth but gave birth to their children in Europe and also remained there during the observation period. Both had a history of treated malaria of unknown species. The respective children were delivered by Cesarean, one because of a treated human immunodeficiency virus (HIV) infection of the mother, the other one as an emergency cesarean section. Being healthy at birth, malaria was diagnosed 5 and 3 weeks post-partum. Detailed information is presented in Table 5.

**Table 5:** Characteristics of severe congenital malaria

Authors, year of publication	Sex	Birth weight (kg)	Previous residence of mother	Country of birth	Diagnostics	Parasitaemia	Body temperature (°C)	Hemoglobin level (g/dl)	Treatment	Outcome	Concomitant medication	Comment
Penazzato et al. [26], 2007	–	3.13	Nigeria	Italy	MIC + PCR	0.01%	–	5.4	Quinine sulphate 20 mg/kg/d for 5 days	Recovered without sequelae	Zidovudine	Mother HIV positive, no materno-foetal transmission of HIV
Jenkins et al. [27], 1957	M	4	East Africa	England	MIC	–	40	6.8	Proguanil 0.25 g daily for 5 days followed by: chloroquine sulphate ¼ tablet twice daily for 2 days, then ¼ tablet daily for 2 days, then ¼ tablet weekly for 3 weeks	Recovered without sequelae	Penicillin, potassium permanganate baths, local aqueous gentian violet 0.66%	Pubic rash after day 3
											Erythromycin	Given on suspicion with proguanil
											Ferrous sulphate	

*MIC* microscopy, *PCR* polymerase chain reaction, *M* male, *–* not mentioned in the original publication

### Relapse

From the description of *P. ovale* as distinct species in 1922 up to 2015 a total of 18 cases with potentially relapsing *P. ovale* parasitaemia according to the inclusion criteria applied for this systematic review were reported in scientific literature. These patients were described to have experienced a total of 28 potential relapse events. 4 cases (22%) occurred in tourists, 14 (78%) in malaria infection studies. Sex was specified in 44% of the patients, all of them were male. Fever was mentioned in five episodes, other clinical information about relapse characteristics was missing. The most commonly used drugs to treat primary infections and relapses were chloroquine and quinine sulfate. Median time between primary infections and first potential relapses was 17 weeks (min–max 2–60 weeks). The median time between first and second potential relapse was also 17 weeks, ranging from 5 to 72 weeks. The time between second and third relapse was not reported. Six relapses occurred despite previous primaquine treatment. Eight individuals presented with two relapses and one individual relapsed three times. Details can be found in Tables 6 and 7.

**Table 6:** Baseline characteristics of potentially relapsing patients

Authors, year of publication	Patient’s status	Age (years)	Sex	Probable origin of infection	Chemoprophylaxis	Parasitaemia (parasites/µl)
Bottieau [28], 2005	T	17	M	Ghana	Mefloquine	–
	T	22	M	Nigeria	Mefloquine	–
	T	14	M	Uganda	None	–
Collins et al. [29], 2002	I	–	–	NA	NA	3780
	I	–	–	NA	NA	2220
	I	–	–	NA	NA	8560
	I	–	–	NA	NA	9810
	I	–	–	NA	NA	5376
Nathwani et al. [30], 1991	T	24	M	Papua New Guinea	Chloroquine, pyrimethamine	–
Chin et al. [31], 1971	E	–	M	NA	NA	–
	E	–	M	NA	NA	–
	E	–	M	NA	NA	–
	E	–	M	NA	NA	–
	E	–	M	NA	NA	–
Garnham et al. [32], 1955	I	–	–	NA	NA	–
	I	–	–	NA	NA	–
Jeffery [33], 1954	I	–	–	NA	NA	–
	I	–	–	NA	NA	–

*T* tourist, *E* sporozoite induced experimental infection, *I* malaria infection therapy in neurosyphilis patients, *M* male, *F* female, *NA* not applicable, – not mentioned in the original publication

**Table 7:** Relapse characteristics

Authors	Diagnostic method primary infection	Treatment primary infection	Dosage primary infection	PQ therapy primary infection?	Time between primary infection and 1st relapse (weeks)	Diagnostic method 1st relapse	Treatment 1st relapse	PQ therapy 1st relapse?	Time between 1st and 2nd relapse (weeks)	Diagnostic method 2nd relapse	Treatment 2nd relapse	PQ therapy 2nd relapse?	Time between 2nd and 3rd relapse (weeks)	Treatment 3rd relapse
Bottieau [28]	MIC	Quinine	1.5 g/day for 5 days	Y, “standard regimen” (presumably 15 mg/day for 14 days)	7	MIC	Chloroquine 1.5 g over 3 days	Y, 7 mg/kg over 3 weeks	NA	NA	NA	NA	NA	NA
		Doxycycline	100 mg/day for 7 days											
	–	Quinine–doxycycline	–	Y, “standard regimen”	2	–	Chloroquine, dosage unknown	Y, 10 mg/kg over 4 weeks	NA	NA	NA	NA	NA	NA
	–	Chloroquine	–	N	60	–	Atovaquone–proguanil, dosage unknown	Y, 5 mg/kg over 6 weeks	78	MIC	Atovaquone–proguanil, then chloroquine	Y, 8 mg/kg over 3 weeks	NA	NA
Collins et al. [29]	MIC	Chloroquine	1.5 g over 3 days	N	22	MIC	Chloroquine, dosage unknown	N	17	MIC	Chloroquine, dosage unknown	–	NA	NA
	MIC	Chloroquine	1.5 g over 3 days	N	10	MIC	–	–	NA	NA	NA	NA	NA	NA
	MIC	Chloroquine	1.5 g over 3 days	N	15	MIC	–	–	NA	NA	NA	NA	NA	NA
	MIC	Chloroquine	1.5 g over 3 days	N	24	MIC	–	–	NA	NA	NA	NA	NA	NA
	MIC	Chloroquine	1.5 g over 3 days	N	20	MIC	–	–	NA	NA	NA	NA	NA	NA
Nathwani et al. [30]	MIC	Chloroquine	15 mg/day for 14 days	Y, 15 mg/day for 14 days	17	MIC	Chloroquine 1.5 g over 3 days	Y, 30 mg/day for 21 days	NA	NA	NA	NA	NA	NA
Chin et al. [31]	MIC	Quinine sulphate	650 mg 8-hourly for 5 days	N	–	MIC	Quinine sulphate 650 mg 8-hourly for 5 days	N	5	MIC	Quinine sulphate 650 mg 8-hourly for 5 days	N	–	Quinine sulphate 650 mg 8-hourly for 5 days
	MIC	Quinine sulphate	650 mg 8-hourly for 5 days	N	2	MIC	Quinine sulphate 650 mg 8-hourly for 5 days	N	20	MIC	Quinine sulphate 650 mg 8-hourly for 5 days	N	NA	NA
	MIC	Quinine sulphate	650 mg 8-hourly for 5 days	N	36	MIC	Quinine sulphate 650 mg 8-hourly for 5 days	N	NA	NA	NA	NA	NA	NA
	MIC	Quinine sulphate	650 mg 8-hourly for 5 days	N	–	MIC	Quinine sulphate 650 mg 8-hourly for 5 days	N	–	MIC	Quinine sulphate 650 mg 8-hourly for 5 days	N	NA	NA
	MIC	Chloroquine	600 mg single dose	N	–	MIC	Chloroquine 600 mg single dose	N	–	MIC	Chloroquine 600 mg single dose	Y, 15 mg/day for 14 days	NA	NA
Garnham et al. [32]	MIC	Chloroquine	–	N	15	MIC	No treatment	N	10	MIC	No treatment	N	NA	NA
	MIC	Chloroquine	–	N	14	MIC	No treatment	N	14	MIC	No treatment	N	NA	NA
Jeffery [33]	MIC	Chloroquine	–	N	21	MIC	Chloroquine, dosage unknown	N	22^a^	MIC	–	–	NA	NA
	MIC	chloroquine	–	N	34	MIC	chloroquine, dosage unknown	Y, dosage unknown	NA^a^	NA	NA	NA	NA	NA

*MIC* microscopy, *Y* yes, *N* no, *NA* not applicable, – not mentioned in the original publication

^a^The patient who did not receive primaquine treatment for his 1st relapse developed a second one, however it was not clear from the article, which one of the two patients developed the described second relapse

Diagnostics relied exclusively on microscopy. PCR correction of the infective species was not performed. Furthermore, there were no articles proving a causal relationship between dormant liver stages and reappearances of *P. ovale* infections in the human host.

## Discussion

Several small literature reviews focusing on specific but limited aspects of *P. ovale* malaria have been previously published, most often appended to case reports. The epidemiology of ovale malaria in a high endemic setting has been demonstrated with long-term surveillance data [34, 35]. *P. ovale* has also been addressed in the context of other infectious diseases [36, 37]. However, to date, the scientific literature does not provide a systematic overview focusing on clinical, therapeutic and relapse characteristics of *P. ovale*. As to the dimorphism of *P. ovale*, too few articles distinguished between the sympatric species to suggest potential differences. This systematic review therefore combines data from both *P. ovale* species.

### Evaluation of current treatment recommendations

Chloroquine has been the recommended treatment for *P. ovale* malaria for many years. In the latest guideline for the treatment of malaria, the WHO strongly recommends to treat *P. ovale* and other non-falciparum Plasmodium species with artemisinin-based combination therapy or chloroquine on the basis of “high-quality evidence”. Following elaborations of underlying studies in the WHO guideline however rather break this down to experience [38]. In this systematic review, no high-quality studies supporting current treatment recommendations were identified. Not a single randomized controlled clinical trial on *P. ovale* malaria has been published in scientific literature. This finding is supported by a report by Visser et al. [37]. Although chloroquine has been tested in small prospective uncontrolled trials, one might question whether this small number of participants and a lack of control groups in all studies provide enough evidence for unequivocal treatment recommendations. Summing up all published reports and clinical experience, it becomes evident that anti-malarial drugs employed for *P. falciparum* are also effective for *P. ovale*. However, scientifically sound evidence for this is currently missing.

### Severe disease

In 1932, James and coworkers stated that it was unlikely that another malignant species besides *P. falciparum* would be discovered [39]. Since then *Plasmodium knowlesi* was found to be infective for humans leading to life-threatening quotidian malaria. Also the previously considered benign malaria species *P. malariae, P. vivax* and *P. ovale* were reported to cause severe disease and even death in a small minority of patients. To date little is known on the specific pathogenesis of severe diseases in these non-falciparum malarias. The results of this systematic review support this understanding.

It is of interest that ARDS was the main feature of severe disease in *P. ovale* malaria as it was described in returning travellers with *P. vivax* malaria [40]. The potential coincidence that the two patients with a history of tuberculosis 10 years and more ago both died from ARDS raises the question whether a preexisting pulmonary condition may be a risk factor for respiratory complications of *P. ovale* infection [41, 42]. Anaemia was also reported as a feature of severe *P. ovale* malaria, however due to its multifactorial aetiology it is difficult to attribute this with confidence to *P. ovale* infection. Nevertheless it has been reported concordantly in paediatric patients with *P. vivax* infection in Asia [43].

An important limitation in the description of severe cases of *P. ovale* infection is the only partly performed molecular assessment of blood samples. Although light microscopy forms the current gold standard for malaria diagnostics, its sensitivity is inferior to most molecular methods. Additionally, species determination and distinction, especially between *P. ovale* and *P. vivax* can be challenging most notably in low parasitaemic smears [44, 45]. It is, therefore, not possible to entirely exclude the possibility of coinfection with other Plasmodium species including *P. falciparum* in these cases.

Congenital malaria is a rare finding in non-endemic countries. Even more surprising was the identification of two cases of congenital *P. ovale* malaria with severe anaemia in Europe. Both mothers had been living in an endemic country in the past. Interestingly, one of the infants was born to a HIV positive mother. An association between HIV and the incidence of *P. falciparum* in pregnancy has already been shown [46] and it might be speculated that the same is true for *P. ovale*. In 2008, Vottier et al. reported another congenital *P. ovale* infection transmitted by an HIV positive mother which was however not severe [47].

### The concept of hypnozoite-induced relapse in *P. ovale* malaria

Although the concept of hypnozoite-induced relapse in all tertian malarias seemed as a unanimous concept until recently, molecular evidence supportive for this model is scarce. A recent experimental study in mice engrafted with human hepatocytes observed uninucleate parasitic structures measuring ~5 µm (day 8) and late schizonts (day 21) after *P. ovale* sporozoite inoculation [48, 49]. The description of these histological structures resembles the findings of Krotoski described for *Plasmodium cynomolgi bastianelli* in Rhesus monkeys (average diameter 4.5 µm) and for *P. vivax* in chimpanzees (approximately 4–5 µm diameter) [50, 51]. However, this analogy does not constitute proof that these uninucleate structures truly represent hypnozoites or rather retarded forms. Furthermore it does not provide evidence for these structures to cause relapse events [48]. Based on this lack of firm experimental evidence and the scarcity of clinical reports a recent perspective article challenged the current concept of *P. ovale* relapse caused by liver hypnozoites proposing a gradual dormancy concept [52].

The presence of dormancies as such can be assumed as data from malaria elimination settings suggests their important role for sustained malaria transmission, along with *P. vivax* [53].

*Plasmodium ovale* hypnozoites have not yet been unequivocally demonstrated in the human host. As evidenced by this systematic review a total of 18 reported cases of *P. ovale* relapse in nearly 100 years do not provide solid evidence for the current relapse theory. On the other hand, experiments and malaria treatment of neurosyphilis patients have shown that in case of repetitive inoculation with the same strain, immunity to this homologous challenge develops fast and subsequent infections remain often asymptomatic [54, 55]. Hence, it may be speculated that a true relapse may lead to mitigated symptoms or may even be sub-clinical.

In this context, it is of interest to note that six potential relapses occurred despite previous primaquine treatment. However, intake of primaquine has not been evaluated in these patients.

Historically, the concept of treatment of *P. ovale* relapses with an 8-aminoquinoline is based on the observation that quinine and pamaquine (the first synthetic 8-aminoquinoline) together were more effective in the treatment of certain malaria cases than quinine alone. When Sinton and Bird observed that pamaquine reduced the relapse rate of *P. vivax* malaria [56] several 8-aminoquinoline derivatives were synthesized and tested for this purpose. Primaquine finally showed a higher anti-relapse effect than pamaquine with reduced toxicity among the most promising substances, but effectiveness for *P. ovale* relapses has since then only been presumed and never demonstrated [57]. Importantly, from a methodological point of view, to prove the effectiveness of a medication it is necessary to first unequivocally demonstrate the existence of the condition to be treated—in this case hypnozoite-induced relapse.

Richter et al. questioned the existence of relapses in *P. ovale* in a review in 2010. They stated that “it may be difficult to differentiate a true relapse from a primary malaria attack with a long latency” [10]. To overcome that difficulty, the analysis was restricted to cases, which did not reside in a malaria endemic area between the occurrences of primary infection and relapse. In addition, the species of the primary infection had to be explicitly mentioned to be *P. ovale* and treated with anti-malarial chemotherapy. Comparing the results of this systematic review with those of Richter et al. [52], these strict criteria are the main reason why even fewer cases of potential relapses were observed here.

Finally, only one potential case of relapse that was investigated with molecular methods could be identified in the literature [58, 59]. As this case occurred in an endemic area, the report did not fit the criteria of this systematic review and was therefore not included in the primary analyses. After personal communication with one of the authors (Fuehrer) the confirmation for this potential relapse case was based on the sequence homology of partial *cox1*, SSU rRNA, and *porbp2* loci between the primary and the potential relapse isolate [60]. These markers are usually not used for intraspecific distinction but for differentiation between the species. The multigene approach, however, enhances the significance of the result. In summary, the identification of highly sensitive genetic markers or techniques that can discriminate between hypnozoite induced relapse and other sources of recurrent infections is still a work in progress.

Limitations of this systematic review are the low strength of evidence of the included studies based on their study design. At the same time, they form the only available evidence to address the review questions and form the basis of current recommendations.

## Conclusion

In conclusion, this review of the scientific literature between 1922 and 2015 did not reveal a single high quality randomized controlled clinical trial. The reported evidence indicates that *P. ovale* is capable of evoking severe disease, severe congenital malaria and even death. Evidence for *P. ovale* related recommendations, however, seems to be scarce and is often based on clinical experience rather than on solid scientific evidence. Accordingly, this underlines the importance for clinical trials with larger sample size to obtain the efficacy of several treatment options for *P. ovale*.

Evidence for relapses in *P. ovale* malaria is poor. Relapses in the human host have so far only once been studied with molecular methods. Hence, there is a need to further explore the *P. ovale* relapse theory and find scientifically sound evidence that proves or disproves the existence of relapses and of hypnozoites as origin of such potential *P. ovale* relapse events. With that knowledge, one might also gain a new perspective on the adequate management for the radical cure of tertian ovale malaria—a neglected malaria, which in the future may gain in public health importance in the setting of successful elimination campaigns for falciparum malaria.

## Additional files

**Additional file 1.** Alphabetic list of included articles with overall quality of reporting and risk of bias assessment. -, not determined; overall risk of bias was declared “high” for case reports and case series; for applicable trials, overall risk of bias results from the detailed risk of bias assessment outlined in the Additional file 2; detailed completeness of reporting assessment is displayed in the Additional file 3.

**Additional file 2.** Detailed risk of bias assessment of prospective uncontrolled clinical trials. Key: 3 or more bias high risk: over all high risk of bias; 3 or more bias medium risk, 2 or less bias high risk: over all medium risk of bias.

**Additional file 3.** Detailed completeness of reporting assessment with a focus on *P. ovale* relevant information. NA, not applicable; CD, cannot be determined; *, for hyper endemic areas adequate length of follow-up was 14 days, otherwise 28 days; ~, total parasitaemia of patients given, else well described; Key: one partial or CD, else yes and NA: good; one no and one partial or CD, else yes: medium; 2-3 times partial and CD, else yes: medium; more than 3 partial and CD: poor; more than 2 no: poor.

## Abbreviations

ARDS: acute respiratory distress syndrome
cox1: cytochrome c oxidase subunit 1
FCT: fever clearance time
HIV: human immunodeficiency virus
MTHFR: methylentetrahydrofolate reductase defect
PCT: parasite clearance time
porbp2: *Plasmodium ovale* reticulocyte binding protein 2
RCT: randomized controlled trial
SSU rRNA: small subunit ribosomal ribonucleic acid
VFR: visiting friends and relatives
WHO: World Health Organization
