# Supplementary material for: A systematic review of the clinical presentation, treatment and relapse characteristics of human Plasmodium ovale malaria
Source: Malar J. 2017 Mar 11;16:112. doi: 10.1186/s12936-017-1759-2 (PMC5346189; doi:10.1186/s12936-017-1759-2)
Supplement: Supplementary file 1 — Additional file 1. Alphabetic list of included articles with overall quality of reporting and risk of bias assessment. -, not determined; overall risk of bias was declared “high” for case reports and case series; for applicable trials, overall risk of bias results from the detailed risk of bias assessment outlined in the Additional file 2; detailed completeness of reporting assessment is displayed in the Additional file 3. [file 12936_2017_1759_MOESM1_ESM.docx]

| **Authors** | **Year of publication** | **Applicable *P.ovale* cases** | **Study design** | **Overall completeness of reporting** | **Overall risk of bias** |
| --- | --- | --- | --- | --- | --- |
| Bock | 1939 | 2 | historical case series | good | high |
| Bottieau et al. | 2005 | 3 | historical case series | good | high |
| Chin et al. | 1971 | 5 | case series | intermediate/good | high |
| Cinquetti et al. | 2010 | 1 | case report | good | high |
| Collins et al. | 2002 | 5 | retrospective cohort study | good | high |
| Coton et al. | 2011 | 1 | case report | good | high |
| Danis et al. | 1982 | 4 | prospective uncontrolled clinical trial | poor | high |
| Facer et al. | 1991 | 1 | case report | good | high |
| Fairley | 1933 | 1 | case report | good | high |
| Filler et al. | 2003 | 1 | notifiable disease register | good | - |
| Garnham et al. | 1955 | 2 | science communication | good | - |
| Hachimi et al. | 2013 | 1 | case report | good | high |
| Haydoura et al. | 2010 | 1 | case report | good | high |
| Jeffery et al. | 1954 | 2 | comparative study | intermediate | high |
| Jenkins | 1957 | 1 | case report | good | high |
| Lahlou et al. | 2012 | 1 | case report | intermediate | high |
| Lau et al. | 2013 | 2 | historical case series | good | high |
| Lee et al. | 1999 | 1 | case report | good | high |
| Lemmerer et al. | 2015 | 1 | case report | good | high |
| Monlun et al. | 1989 | 1 | case report with attached historical case series | good | high |
| Nathwani et al. | 1991 | 1 | case report | good | high |
| Patel | 1993 | 1 | case report | intermediate | high |
| Penazzato | 2007 | 1 | case report | good | high |
| Radloff et al. | 1996 | 3 | prospective uncontrolled clinical trial | intermediate | high |
| Ringwald et al. | 1997 | 10 | prospective uncontrolled clinical trial | good | medium |
| Rojo-Marcos et al. | 2014 | 3 | retrospective comparative multicenter study | good | low |
| Rojo-Marcos et al. | 2008 | 1 | case report | good | high |
| Roze et al. | 2011 | 1 | case report | intermediate | high |
| Rubinstein et al. | 2005 | 1 | case report | good | high |
| Same-Ekobo et al. | 1999 | 30 | prospective uncontrolled clinical trial | good | medium |
| Siswantoro et al. | 2011 | 11 | prospective uncontrolled clinical trial | good | medium |
| Strydom et al. | 2014 | 1 | case report | good | high |
| Tomar et al. | 2015 | 1 | case report | good | high |
