# Supplementary material for: A systematic review of the clinical presentation, treatment and relapse characteristics of human Plasmodium ovale malaria
Source: Malar J. 2017 Mar 11;16:112. doi: 10.1186/s12936-017-1759-2 (PMC5346189; doi:10.1186/s12936-017-1759-2)
Supplement: Supplementary file 2 — Additional file 2. Detailed risk of bias assessment of prospective uncontrolled clinical trials. Key: 3 or more bias high risk: over all high risk of bias; 3 or more bias medium risk, 2 or less bias high risk: over all medium risk of bias. [file 12936_2017_1759_MOESM2_ESM.docx]

| **Authors** | **Year of publication** | **Selection bias** | **Performance bias** | **Detection bias** | **Attrition bias** | **Reporting bias** | **Other bias** |
| --- | --- | --- | --- | --- | --- | --- | --- |
| Collins et al. | 2002 | high risk: no designated in- and exclusion criteria, uniform strategy for applying intervention | high risk: no blinding of participants and personnel, fidelity to the intervention protocol cannot be determined, concurrent interventions or exposures were not determined; | medium risk: no information about blinding of outcome assessors, exposures and outcomes were assessed consistently; | high risk: no information whether attrition was a concern, also no information on dealings with incomplete outcome data; | low risk: objectives defined and reported |  |
| Danis et al. | 1982 | high risk: uncontrolled trial, no inclusion or exclusion criteria described; | high risk: no blinding of participants and personnel, intervention protocol as such not present; | high risk: no information about blinding of outcome assessors, exposures and outcomes were presented consistently; | high risk: no information whether attrition was a concern, also no information on dealings with incomplete outcome data; | high risk: scarce definition of objectives; | sampling bias: high risk: small sample size |
| Jeffery et al. | 1954 | high risk: no designated in- and exclusion criteria, no randomization of intervention; | high risk: no blinding of participants and personnel, , intervention protocol as such not present; | medium risk: no information about blinding of outcome assessors, outcomes were assessed elaborately; | high risk: no information whether attrition was a concern, also no information on dealings with incomplete outcome data; | medium risk: objectives not defined in detail, objectives were reported |  |
| Radloff et al. | 1996 | medium risk: uncontrolled trial, in- and exclusion criteria applied uniformly; | medium risk: no blinding of participants and personnel, accuracy to intervention protocol was maintained; | medium risk: no blinding of outcome assessors, but interventions, exposures and outcomes were assessed consistently; | high risk: no information whether attrition was a concern, also no information on dealings with incomplete outcome data; | high risk: scarce definition of objectives, therefore no information on completeness of reporting; | sampling bias: high risk: small sample size |
| Ringwald et al. | 1997 | medium risk: internal control without randomization, in- and exclusion criteria applied uniformly; | medium risk: no blinding of participants and personnel, accuracy to intervention protocol was maintained; | medium risk: no blinding of outcome assessors but interventions, exposures and outcomes were assessed consistently; same length of follow up in the groups; | low risk: data of patient lost to follow up was excluded from analyses; | low risk: prespecified outcomes reported; | sampling bias: high risk: small sample size |
| Rojo-Marcos et al. | 2014 | medium risk: country wide submission of applicable samples to the reference center; poor quality of stored samples affected includability; | low risk: concurrent interventions and exposure were taken into account | medium risk: no blinding; interventions, exposures and outcomes were assessed consistently | low risk: one loss to follow up before data ascertainment; | low risk: prespecified outcomes reported; |  |
| Same-Ekobo et al. | 1999 | medium risk: misleading description – described as randomized, non-comparative clinical trial without placebo, still in- and exclusion criteria applied uniformly; | medium risk: no blinding of participants and personnel, accuracy to intervention protocol was maintained; | medium risk: no blinding of outcome assessors, but interventions, exposures and outcomes were assessed consistently; | high risk: no information whether attrition was a concern, also no information on dealings with incomplete outcome data; | low risk: prespecified outcomes reported; | sampling bias: high risk: small sample size |
| Siswantoro et al. | 2011 | medium risk: uncontrolled trial, in- and exclusion criteria applied uniformly; | medium risk: no blinding of participants and personnel, accuracy to intervention protocol was maintained; | medium risk: no blinding of outcome assessors, but interventions,; | low risk: data was censored for patients with incomplete treatment or lost to follow up; | low risk: prespecified outcomes reported; | sampling bias: high risk: small sample size |
