# Supplementary material for: A systematic review of the clinical presentation, treatment and relapse characteristics of human Plasmodium ovale malaria
Source: Malar J. 2017 Mar 11;16:112. doi: 10.1186/s12936-017-1759-2 (PMC5346189; doi:10.1186/s12936-017-1759-2)
Supplement: Supplementary file 3 — Additional file 3. Detailed completeness of reporting assessment with a focus on P. ovale relevant information. NA, not applicable; CD, cannot be determined; *, for hyper endemic areas adequate length of follow-up was 14 days, otherwise 28 days; ~, total parasitaemia of patients given, else well described; Key: one partial or CD, else yes and NA: good; one no and one partial or CD, else yes: medium; 2-3 times partial and CD, else yes: medium; more than 3 partial and CD: poor; more than 2 no: poor. [file 12936_2017_1759_MOESM3_ESM.docx]

| **Authors** | **Year of publication** | **Study design** | **Study question/ objective clearly defined** | **Study population fully described** | **Intervention clearly described** | **Adequate length of follow-up*** | **Results well described** |
| --- | --- | --- | --- | --- | --- | --- | --- |
| Bock | 1939 | historical case series | Yes | Partial | Yes | CD | Yes |
| Bottieau et al. | 2005 | historical case series | Yes | Yes | Yes | Yes | Yes |
| Chin et al. | 1971 | case series | Yes | Partial | Yes | Yes | Partial |
| Cinquetti et al. | 2010 | case report | Yes | Yes | Yes | Yes | Yes |
| Collins et al. | 2002 | retrospective cohort study | Yes | Partial | Yes | NA | Yes |
| Coton et al. | 2011 | case report | Yes | Yes | Yes | Yes | Yes |
| Danis et al. | 1982 | prospective uncontrolled clinical trial | Yes | Partial | Partial | CD | Partial |
| Facer et al. | 1991 | case report | Yes | Yes | NA | NA | Yes |
| Fairley | 1933 | case report | Yes | Yes | Yes | No | Yes |
| Filler et al. | 2003 | notifiable disease register | Yes | Yes | Yes | NA | Yes |
| Garnham et al. | 1955 | science communication | Yes | No | Yes | Yes | Yes |
| Hachimi et al. | 2013 | case report | Yes | Yes | Yes | NA | Yes |
| Haydoura et al. | 2010 | case report | Yes | Yes | Yes | Yes | Yes |
| Jeffery et al. | 1954 | comparative study | Yes | No | Partial | Yes | Yes |
| Jenkins | 1957 | case report | Yes | Yes | Yes | Yes | Yes |
| Lahlou et al. | 2012 | case report | Yes | Yes | Partial | NA | Partial |
| Lau et al. | 2013 | historical case series | Yes | Yes | Yes | Yes | Yes |
| Lee et al. | 1999 | case report | Yes | Yes | Yes | Yes | Yes |
| Lemmerer et al. | 2015 | case report | Yes | Yes | Yes | No | Yes |
| Monlun, et al. | 1989 | case report with attached historical case series | Yes | Yes | Yes | CD | Yes |
| Nathwani et al. | 1991 | case report | Yes | Yes | Yes | Yes | Yes |
| Patel | 1993 | case report | Yes | Yes | Yes | No | Partial |
| Penazzato | 2007 | case report | Yes | Yes | Yes | Yes | Yes |
| Radloff et al. | 1996 | prospective uncontrolled clinical trial | Yes | Partial | Partial | Yes | Partial |
| Ringwald et al. | 1997 | prospective uncontrolled clinical trial | Yes | Yes | Yes | Yes | Yes |
| Rojo-Marcos et al. | 2014 | retrospective comparative multicenter study | Yes | Yes | Yes | CD | Yes |
| Rojo-Marcos et al. | 2008 | case report | Yes | Yes | Yes | Yes | Yes |
| Roze et al. | 2011 | case report | Yes | Yes | Partial | CD | Partial |
| Rubinstein et al. | 2005 | case report | Yes | Yes | Yes | Yes | Yes |
| Same-Ekobo et al. | 1999 | prospective uncontrolled clinical trial | Yes | Yes | Yes | Yes | Partial^~^ |
| Siswantoro et al. | 2011 | prospective uncontrolled clinical trial | Yes | Yes | Yes | Yes | Yes |
| Strydom et al. | 2014 | case report | Yes | Yes | Yes | CD | Yes |
| Tomar et al. | 2015 | case report | Yes | Yes | Partial | CD | Yes |
